# Culturing human intestinal stem cells for regenerative applications in the treatment of inflammatory bowel disease

**DOI:** 10.15252/emmm.201607260

**Published:** 2017-03-10

**Authors:** Fredrik EO Holmberg, Jakob B Seidelin, Xiaolei Yin, Benjamin E Mead, Zhixiang Tong, Yuan Li, Jeffrey M Karp, Ole H Nielsen

**Affiliations:** ^1^ Department of GastroenterologyHerlev HospitalUniversity of CopenhagenHerlevDenmark; ^2^ Division of BioEngineering in MedicineDepartment of MedicineCenter for Regenerative TherapeuticsBrigham and Women's HospitalCambridgeMAUSA; ^3^ Harvard Medical SchoolBostonMAUSA; ^4^ Harvard Stem Cell InstituteCambridgeMAUSA; ^5^ Harvard ‐ MIT Division of Health Sciences and TechnologyCambridgeMAUSA; ^6^ David H. Koch Institute for Integrative Cancer ResearchMITCambridgeMAUSA; ^7^ Broad Institute of Harvard and MITCambridgeMAUSA

**Keywords:** inflammatory bowel disease, intestinal stem cells, organoids, regenerative medicine, support matrix, Digestive System, Immunology, Stem Cells

## Abstract

Both the incidence and prevalence of inflammatory bowel disease (IBD) is increasing globally; in the industrialized world up to 0.5% of the population are affected and around 4.2 million individuals suffer from IBD in Europe and North America combined. Successful engraftment in experimental colitis models suggests that intestinal stem cell transplantation could constitute a novel treatment strategy to re‐establish mucosal barrier function in patients with severe disease. Intestinal stem cells can be grown *in vitro* in organoid structures, though only a fraction of the cells contained are stem cells with regenerative capabilities. Hence, techniques to enrich stem cell populations are being pursued through the development of multiple two‐dimensional and three‐dimensional culture protocols, as well as co‐culture techniques and multiple growth medium compositions. Moreover, research in support matrices allowing for efficient clinical application is in progress. *In vitro* culture is accomplished by modulating the signaling pathways fundamental for the stem cell niche with a suitable culture matrix to provide additional contact‐dependent stimuli and structural support. The aim of this review was to discuss medium compositions and support matrices for optimal intestinal stem cell culture, as well as potential modifications to advance clinical use in IBD.

**Glossary**
Anoikis: Dissociation‐induced apoptosis occurring when anchorage‐dependent cells, such as epithelial cells, detach from the underlying extracellular basement membrane. Cell–cell contact can sometimes prevent anoikis from occurring.
Inflammatory bowel disease (IBD): A group of chronic remitting inflammatory conditions localized to the intestine, often debuting in adolescence. The two major subtypes are ulcerative colitis and Crohn's disease, but it also includes microscopic colitis and diversion colitis. Crohn's disease can affect segments of the entire gastrointestinal tract, while ulcerative colitis is restricted to the colon. Symptoms include abdominal pain, diarrhea, anemia, rectal bleeding, and weight loss. However, the condition is often complicated by extra‐intestinal symptoms, commonly affecting skin, joints, or eyes. IBD is frequently treated with anti‐inflammatory and immunomodulatory drugs, although surgical bowel resection may be required in severe disease.
Intestinal organoid: A three‐dimensional organlike structure grown *in vitro*, consisting of intestinal epithelial cells. The nomenclature varies and is also referred to as a mini‐gut. It has been suggested that the term organoid should be reserved for structures containing both epithelial and mesenchymal components. In turn, enteroids may be used for structures consisting solely of epithelial components.
Intestinal stem cell niche: A specific microenvironment which dynamically regulates stem cell renewal and differentiation. It consists of an intricate signaling system of chemical mediators and mechanical cues derived from epithelial and mesenchymal sources, as well as from the extracellular matrix.

## Introduction

Inflammatory bowel disease (IBD) of which Crohn's disease (CD) and ulcerative colitis (UC) are the two most prevalent entities, constitute a chronic remitting disorder with increasing incidence worldwide, reported in the range of up to 50 per 100,000 in the Western population (Molodecky *et al*, 2012). IBD causes lifelong morbidity, including extra‐intestinal complications (Larsen *et al*, 2010), and can greatly impair quality of life of affected individuals. It also constitutes a considerable economic burden for society in terms of direct medical costs (Burisch *et al*, 2013), and indirect costs arising from impaired work performance, including sick leave (Hoivik *et al*, 2013).

Mucosal healing is associated with a more favorable prognosis for patients with IBD, including lower relapse and hospitalization rates, as well as a diminished risk for surgery (Peyrin‐Biroulet *et al*, 2011; Shah *et al*, 2016).

Successful transplantation of intestinal stem cells (ISCs), which are responsible for tissue homeostasis and injury response, has been achieved in murine models of experimental colitis, demonstrating that they adhere to and become an integrated part of the epithelium, thereby improving mucosal healing (Yui *et al*, 2012; Fordham *et al*, 2013; Fukuda *et al*, 2014). Hence, ISC transplantation might constitute an appealing therapeutic approach to re‐establish the epithelial barrier in IBD.

ISCs are located at the base of the intestinal crypts where they renew the epithelium through differentiation to multiple epithelial progenies (Bjerknes & Cheng, 2006), and drive mucosal regeneration. Several genes mark the ISC population, including *LGR5* (Barker *et al*, 2007), olfactomedin 4 *(OLFM4)* (van der Flier *et al*, 2009a), and *ASCL2* (van der Flier *et al*, 2009b).

ISCs can be cultured *in vitro*, giving rise to three‐dimensional self‐organizing structures called organoids (Sato *et al*, 2009). Organoids resemble the intestinal epithelium *in vivo,* possessing crypt and villus domains that contain multiple epithelial cell types derived from the ISCs (Sato *et al*, 2011b).

Since intestinal stemness is determined by extrinsic signals, multiple culture protocols exist to emulate the *in vivo* ISC niche*,* and to sustain them *in vitro*. Protocols for human cell culture are based on a coordinated stimulation of wingless‐type mouse mammary tumor virus integration site (WNT) signaling, epidermal growth factor (EGF), as well as inhibition of bone morphogenic protein (BMP), transforming growth factor‐β (TGF‐β) signaling, and p38 signaling (Jung *et al*, 2011; Sato *et al*, 2011a). The primary distinguishing factors between protocols are the growth medium constituents and the support matrices applied, resulting in differences in cellular composition. Nevertheless, most culture protocols for human intestinal organoids are unable to efficiently increase the frequency of ISCs within organoid structures, as only a few percent of the cells contained are self‐renewing and multipotent stem cells (Jung *et al*, 2011). This raises the need for devising improved culture techniques to yield a purer population of ISCs, applicable for clinical transplantation strategies.

This review provides an updated overview of current growth protocols for human ISCs *in vitro,* seeking to pinpoint obstacles in stem cell enrichment and matrix support, which should be addressed to allow for regenerative application of ISCs in IBD.

## Growth medium

The basal medium for culturing ISCs often contains Advanced Dulbecco's Modified Eagle Medium/F12, supplemented with Glutamax, B‐27, N‐2, HEPES, acetylcysteine, and penicillin/streptomycin, though human colonic organoids can be sustained without N‐2 supplement (Fujii *et al*, 2015). It is also possible to replace B‐27, N‐2, and acetylcysteine with serum (Van Dussen *et al*, 2015), but this approach may pose other challenges for clinical applications, as discussed in the subsequent section. The basal medium prevents bacterial contamination and provides buffering capacity, necessary amino acids, vitamins, antioxidants, hormones as well as inorganic compounds.

Apart from the basic components, the growth media applied may vary according to the type or composition of growth factors and small molecules, either in the form of conditioned media, or high‐purity recombinant proteins. Frequently used growth media constituents, their working mechanisms and effects, as well as applications are summarized in Table 1.

**Table 1:** Frequently used growth media constituents, their working mechanisms and effects, as well as applications

Growth medium constituents	Working mechanism in ISCs	Effect on ISCs and application
WNT3a^a^	Activates canonical WNT signaling (Clevers & Nusse, 2012)	Stimulates crypt cells proliferation and maintains the stem cell state (Clevers & Nusse, 2012; Farin *et al*, 2012; Krausova & Korinek, 2014)
R‐spondin 1^a^	Augments WNT/β‐catenin signaling (de Lau *et al*, 2014)	Stimulates crypt cell proliferation and maintains stem cell state (Farin *et al*, 2012; Krausova & Korinek, 2014; de Lau *et al*, 2014)
CHIR99021	Stimulates canonical WNT signaling (Yin *et al*, 2014)	Stimulates stem cell proliferation and can be used in combination with VPA, when growing single mouse ISCs in absence of Paneth cells (Yin *et al*, 2014)
Valproic acid	Inhibits histone deacetylase and activates Notch signaling (Yin *et al*, 2014)	Maintains proliferative crypts and blocks secretory differentiation (Sato *et al*, 2011b). Can be used in combination with CHIR99021 when growing single mouse ISCs in absence of Paneth cells (Yin *et al*, 2014)
Noggin^a^	Inhibits BMP signaling (Haramis *et al*, 2004)	Stimulates crypt formation (Haramis *et al*, 2004)
Jagged‐1	Activates Notch signaling (Sato *et al*, 2009)	Maintains the stem cell state, and promotes proliferation, while blocking secretory differentiation, thereby maintaining proliferative crypts (Stanger *et al*, 2005; Van Dussen *et al*, 2012) Used in the early phase of single‐cell cultures in absence of Notch signaling from adjacent supportive cells (Sato *et al*, 2009; Grabinger *et al*, 2014)
EGF^a^	Activates RAS/RAF/MEK/ERK signaling pathway (Suzuki *et al*, 2010; Date & Sato, 2015)	Stimulates stem cell migration, proliferation, and inhibits apoptosis (Frey *et al*, 2004; Suzuki *et al*, 2010)
PGE_2_	Enhances canonical WNT signaling (Buchanan & DuBois, 2006)	Prevents anoikis as well as promotes stem cell survival and proliferation, thereby improving culture efficiency. Stimulates spheroid morphology (Cohn *et al*, 1997; Joseph *et al*, 2005)
Nicotinamide	Inhibits the activity of sirtuins (Denu, 2005)	Improves ISC maintenance when cultured > 1 week (Sato *et al*, 2011a). Often used for long‐term human intestinal organoid cultures (Sato *et al*, 2011a), but can be omitted (Fujii *et al*, 2015)
Gastrin‐17	Not decisively concluded	Marginally increases culture efficiency (Sato *et al*, 2011a)
A83‐01 or SB431542^a^	Inhibits TGF‐β signaling (Sato *et al*, 2011a)	Inhibits differentiation and allows human intestinal stem cell cultures to be sustained in the long term (Sato *et al*, 2011a)
SB202190^a^	Inhibits P38 MAPK (Sato *et al*, 2011a)	Inhibits secretory differentiation, increases plating efficiency, and decreases degradation of the EGF receptor (Frey *et al*, 2006; Sato *et al*, 2011a; Date & Sato, 2015). Allows human intestinal stem cell cultures to be sustained in the long term (Sato *et al*, 2011a)
Y‐27632 or thiazovivin	Inhibition of caspase‐3 (Wu *et al*, 2015)	Prevents anoikis after single‐cell dissociation (Watanabe *et al*, 2007). Used in the early phase of single‐cell cultures
IL‐22	JAK/STAT signaling (Lindemans *et al*, 2015)	ISC proliferation and organoid growth. Can potentially further increase ISC expansion and make EGF redundant (Lindemans *et al*, 2015)

a Mandatory growth medium components for long‐term culturing human intestinal stem cells as organoids.

## WNT/R‐spondin signaling

WNT signaling plays a crucial role in tissue development and homeostasis, though over‐activity is associated with tumorigenesis (Krausova & Korinek, 2014).

Two primary branches of WNT signaling exist: canonical and non‐canonical. Non‐canonical signaling is implicated in the establishment of cell polarity and migration, as well as inflammation and cancer development (Kumawat & Gosens, 2016), and has been less implicated in sustaining ISCs.

**Figure 1:**
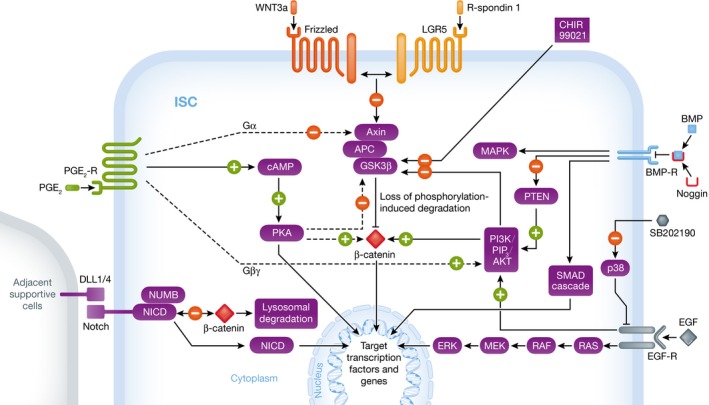
Suggested downstream effects of growth medium components on canonical WNT signaling Activation of the WNT pathway inhibits phosphorylation‐induced degradation of β‐catenin mediated by Axin/APC/GSK3β, which precipitates nuclear translocation of β‐catenin and activation of target genes. BMP inhibition and EGF activation increase nuclear β‐catenin levels, due to phosphorylation and inactivation of GSK3β or phosphorylation of β‐catenin itself. Similarly, CHIR99021 can increase WNT signaling by inactivation of GSK3β. PGE_2_ can promote β‐catenin stability through suppression of GSK3β, but perhaps also through interaction between PGE_2_‐R subunits and Axin, activation of cAMP/PKA and PI3K/PIP_3_/AKT activity. SB202190 inhibits p38, thereby decreasing ligand‐driven degradation of the EGF receptor. Delta like canonical Notch ligand 1/4 (DLL1/4) can activate membrane‐bound Notch, and the adaptor protein NUMB can associate with unphosphorylated β‐catenin, precipitating its lysosomal degradation, thereby dampening WNT activity.

The canonical WNT pathway is β‐catenin dependent, and it is best studied owing to its essential role in preserving the undifferentiated stem cell state and promoting proliferation (van de Wetering *et al*, 2002). The canonical WNT pathway is activated by binding of a WNT ligand to the Frizzled receptor and its co‐receptor complex low‐density lipoprotein receptor‐related protein 5/6 (LRP5/6). This leads to stabilization of β‐catenin that translocates to the nucleus where it interacts with T‐cell factor/lymphoid enhancer factor (TCF/LEF), thereby activating downstream target genes such as *c‐MYC, Cyclin D1,* and *Axin2* (Mah *et al*, 2016). In the absence of WNT activation, β‐catenin is subject to proteosomal degradation promoted by the Axin/APC/GSK3β complex‐mediated phosphorylation. WNT signaling can in turn be augmented by binding of R‐spondins (RSPOs) to the LGR5 receptor, which suppresses internalization and degradation of Frizzled by neutralizing transmembrane ligases RNF43/ZNRF3 (Li *et al*, 2012). Several other signaling pathways, for example, BMP, Notch, EGF, and prostaglandin E_2_ (PGE_2_), have been suggested to interact with the canonical WNT pathway as summarized in Fig 1.

To culture human intestinal organoids, the growth medium needs to be supplemented with a WNT ligand, and conditioned medium is often applied. The use of conditioned media is generally more cost‐effective than recombinant proteins, though conditioned media contains serum for the purpose of protein stabilization, and includes the inherent risk for xenogeneic and pathogenic contamination, although presumably quite small (Tekkatte *et al*, 2011). Serum also contains undefined components and demonstrates batch‐to‐batch variability that hampers standardization. Nonetheless, mesenchymal stem cells cultured in serum have already been used in human trials without issues (Panes *et al*, 2016). Nevertheless, serum substitutes have successfully been applied to circumvent potential issues when culturing human mesenchymal stem cells (Kim *et al*, 2013).

Human recombinant WNT3a is commercially available, but substituting conditioned medium with recombinant WNT3a reduces the growth efficiency of intestinal organoids (Fujii *et al*, 2015). WNT proteins are palmitoylated, which is crucial for interactions with the Frizzled receptor, though this is difficult to express and to purify (Willert *et al*, 2003). Impurities can activate mediators of TGF‐β and BMP signaling, which is undesirable when culturing ISCs (Carthy *et al*, 2016). Even though human high‐purity recombinant WNT3a has become commercially available, it is unlikely to be a fitting substitute for WNT3a conditioned medium, since purified WNT proteins rapidly lose their biologic activity, presumably due to hydrophobic aggregation (Dhamdhere *et al*, 2014). However, it was recently shown that the serum glycoprotein afamin stabilizes WNT proteins by forming water‐soluble complexes, thereby preventing aggregation while at the same time maintaining their biologic activity (Mihara *et al*, 2016). This is reflected in the EC_50_ value that is estimated to be 5–10 times lower for afamin/WNT3a versus purified WNT3a. Hence, afamin/WNT3a complex might be a better means to accomplish WNT activation in ISC‐derived organoids for clinical applications.

Small molecules such as the GSK3β inhibitor CHIR99021, which prevents β‐catenin degradation, can further activate the WNT pathway (Yin *et al*, 2014).

Augmentation of WNT signaling with RSPO1 is most commonly used, either in the form of conditioned media or as a recombinant protein, with similar efficacy in human organoid growth (Fujii *et al*, 2015).

## BMP and TGF‐β signaling

BMP signaling gradients promote spatially arranged differentiation of ISCs, in part by suppressing WNT signaling, thereby regulating the number of stem cells *in vivo* (He *et al*, 2007; Krausova & Korinek, 2014).

BMP signaling is activated by ligand binding to a multi‐component receptor complex and incorporates several complex pathways, for example, activation of the SMAD cascade (SMAD 1, 5, and 8), and MAPK, as well as positive regulation of PTEN (He *et al*, 2007; Katagiri & Watabe, 2016). In turn, PTEN negatively regulates the phosphatidylinositol 3‐kinase (PI3K)/phosphatidylinositol triphosphate (PIP_3_)/AKT cascade, which has several downstream substrates, including GSK3β and β‐catenin (He *et al*, 2007). Thus, AKT interacts with the canonical WNT pathway by increasing β‐catenin levels in the nucleus due to phosphorylation and inactivation of GSK3β or phosphorylation of β‐catenin itself (Fig 1). Hence, active BMP signaling suppresses the β‐catenin/WNT pathway, thereby counteracting the proliferative effects of WNT activation.

Noggin is a BMP antagonist, and as such, the addition of recombinant Noggin or conditioned medium, combined with exogenous WNT activation, leads to preservation and proliferation of ISCs. Without Noggin, intestinal organoids cannot be maintained in culture (Sato *et al*, 2009).

The TGF‐β pathway activates the SMAD 2/3 cascade, but it clearly demonstrates context dependency (Hata & Chen, 2016), and is capable of activating several other pathways, including the MAPK pathway. The exact mechanism of action in ISCs remains obscure, but TGF‐β appears not to affect ISC proliferation, although it controls clone expansion and extinction, as well as modulates the differentiation of secretory lineage precursors (Fischer *et al*, 2016).

TGF‐β receptor inhibitors, like A83‐01 or SB431542, increase plating efficiency and are necessary for long‐term culture of intestinal organoids by maintaining the undifferentiated stem cell state (Sato *et al*, 2011a).

## EGF

EGF is an important regulator of intestinal epithelial cell migration and proliferation (Suzuki *et al*, 2010). Binding of EGF to its receptor results in induction of tyrosine kinase activity, with subsequent activation of the RAS/RAF/MEK/ERK signaling as well as the PI3K/PIP_3_/AKT cascades, inducing organoid growth (Date & Sato, 2015). The PI3K/PIP_3_/AKT pathway overlaps with the EGF and the BMP pathways, and provides a link to the canonical WNT pathway, as shown in Fig 1.

EGF in the form of recombinant protein is essential for culturing human intestinal organoids, and lack of EGF or addition of an inhibitor of the EGF receptor causes decreased organoid formation and survival (Matano *et al*, 2015). Yet, human intestinal organoids have been cultured without EGF when large amounts of serum were used, in the form of conditioned medium containing WNT, RSPO3, and Noggin (Van Dussen *et al*, 2015).

EGF signaling *in vivo* is partly regulated by a negative feedback system, constituted by the p38 MAPK pathway that affects EGF receptor (Frey *et al*, 2006). This pathway regulates numerous cell responses, including inflammation, apoptosis, cell cycle, differentiation, proliferation, and tumorigenesis (Zarubin & Han, 2005). In the intestinal epithelium, p38 determines whether EGF stimulation results in migration or in proliferation (Frey *et al*, 2004). Pharmacological inhibition of p38 decreases ligand‐driven degradation of the EGF receptor, without affecting its internalization (Frey *et al*, 2006), resulting in increased proliferation. Similarly, deletion of *p38* in intestinal epithelial cells results in increased proliferation, but also in a decreased goblet cell differentiation (Otsuka *et al*, 2010). Hence, a p38 inhibitor, such as SB202190, should be added to the growth medium of intestinal organoids to stimulate proliferation and long‐term maintenance of human ISCs.

IGF‐1 can, similarly to EGF, stimulate PI3K/PIP_3_/AKT and RAS/RAF/MEK/ERK signaling, resulting in growth of intestinal organoids. However, EGF tends to more efficiently induce budding, corresponding to crypt formation and organoid expansion (Reynolds *et al*, 2014).

## Notch signaling

Notch is essential to maintain the ISC pool by controlling stem cell self‐renewal, as well as the balance between absorptive and secretory cell lineage specification (Demitrack & Samuelson, 2016). Pathway inhibition reduces ISCs proliferation and induces secretory lineage differentiation, thereby diminishing the ISC population (van Es *et al*, 2005; Van Dussen *et al*, 2012). Conversely, activation of the Notch pathway maintains stem cell multipotency and promotes stem cell proliferation, while directing progenitors toward an absorptive, rather than a secretory fate (Stanger *et al*, 2005; Demitrack & Samuelson, 2016).

When a Notch ligand binds to the receptor, the Notch intracellular domain (NICD) is separated through proteolytic cleavage, initiating nuclear translocation and activation of target genes (Date & Sato, 2015). However, in some cases, ligand binding is insufficient to cause cleavage and receptor activation. The process requires both ligand stabilization and mechanical force, inducing conformational changes of the receptor (Varnum‐Finney *et al*, 2000; Musse *et al*, 2012). Thus, direct activation of Notch pathway using recombinant Notch ligand has shown limited success.

Genetic activation of the Notch pathway in murine ISCs antagonizes and titrates canonical WNT signaling activity, thereby maintaining the stem cell state and balancing the differentiation process (Tian *et al*, 2015). Similarly, membrane‐bound Notch and its adaptor protein NUMB in human embryonic stem cells and human colon cancer cells associate with unphosphorylated β‐catenin, precipitating its lysosomal degradation (Kwon *et al*, 2011). The process appears to be independent of NICD, as depicted in Fig 1.

When culturing and mechanically passaging intestinal organoids, Notch stimulation is supplied by adjacent supportive cells (Sasaki *et al*, 2016), hence further stimulation is likely redundant. However, when growing dissociated single ISCs attained through enzymatic organoid dissociation, Notch signaling should be stimulated. One common approach is to add Jagged‐1 peptide to the support matrix for the first couple of days (Sato *et al*, 2009; Yin *et al*, 2014), although additional studies are required to demonstrate an increased efficacy. When growing pure murine stem cell cultures, Notch stimulation can be provided by exogenous supplementation of the histone deacetylase inhibitor; valproic acid (VPA) (Yin *et al*, 2014). In terms of clinical applications, VPA has the benefit of already being approved by both EMA and FDA for treatment of epilepsy and certain bipolar disorders, which might simplify the approval process for its application in clinical stem cell enrichment.

## Prostaglandin E_2_

The physiologically active lipid PGE_2_ is produced from arachidonic acid in cell membranes via the cyclooxygenase pathway and binds to a number of G‐coupled cell receptors. PGE_2_ promotes ISC expansion and cell proliferation *in vitro* (Fan *et al*, 2014), inducing organoid swelling and spheroid morphology rather than an organoid crypt structure (Fordham *et al*, 2013). The swelling was recently revealed to be caused by induction of anion and fluid secretion into the organoid lumen (Fujii *et al*, 2016).

PGE_2_ upregulates several WNT target genes (Fan *et al*, 2014), which presumably explains its association with the development of colorectal cancer (Buchanan & DuBois, 2006). It also appears to suppress enterocyte differentiation and to promote repair of the intestinal epithelium (Miyoshi *et al*, 2017).

Studies of ISCs (Miyoshi *et al*, 2017) and vertebrate hematopoietic stem cells (Goessling *et al*, 2009) have revealed that PGE_2_ signaling affects β‐catenin stability through suppression of GSK3β. Several other pathways are suggested to be involved, for example, interaction between PGE_2_ receptor‐subunits and Axin, activation of cAMP/PKA as well as PI3K/PIP_3_/AKT activity (Fig 1) (Evans, 2009). Additionally, PGE_2_ upregulates LGR5 protein in human colorectal adenomas through a β‐catenin independent pathway, a central mechanism in colorectal tumorigenesis (Al‐Kharusi *et al*, 2013). However, data attained from cancer research cannot be extrapolated directly to normal ISCs, since cancer cells might contain mutations of the WNT or PGE_2_ pathways.

PGE_2_ has the benefit of already being approved for clinical use by both EMA and FDA for induction of labor.

## Other small molecules and cytokines affecting intestinal stem cell maintenance

To sustain human ISCs, the vitamin nicotinamide is often added to the growth medium (Sato *et al*, 2011a). Nicotinamide impedes sirtuin activity involved in apoptosis, aging, differentiation, and transcription regulation (Denu, 2005). However, nicotinamide can be omitted without affecting long‐term sustainability of human colonic stem cells (Fujii *et al*, 2015).

When culturing dissociated single stem cells, the Rho‐associated protein kinase (ROCK) inhibitors, Y‐27632 (Watanabe *et al*, 2007) or thiazovivin (Wang *et al*, 2013), can be added to the growth medium for the first few days to prevent anoikis. Research on pluripotent stem cells has suggested that ROCK inhibitors suppress caspase‐dependent cell death (Wu *et al*, 2015).

Amidated gastrin‐17 is regularly used when culturing intestinal organoids, though it only marginally improves culture efficiency and it may therefore be omitted (Sato *et al*, 2011a).

Addition of the cytokine interleukin 22 (IL‐22) to the growth medium has shown to increase the proliferation of ISCs and to cause EGF redundancy when culturing human intestinal organoids (Lindemans *et al*, 2015). It activates STAT3, which causes growth of human intestinal organoids independent of Paneth cells as well as both Notch and WNT signaling (Lindemans *et al*, 2015).

Many of the small molecules used to culture ISCs are available in high‐purity formulations, though safety data are sparse, which could provide translational limitations. However, very low concentrations of the small molecules are used in culture and can presumably be washed off prior to transplantation.

## Culture matrices

**Figure 2:**
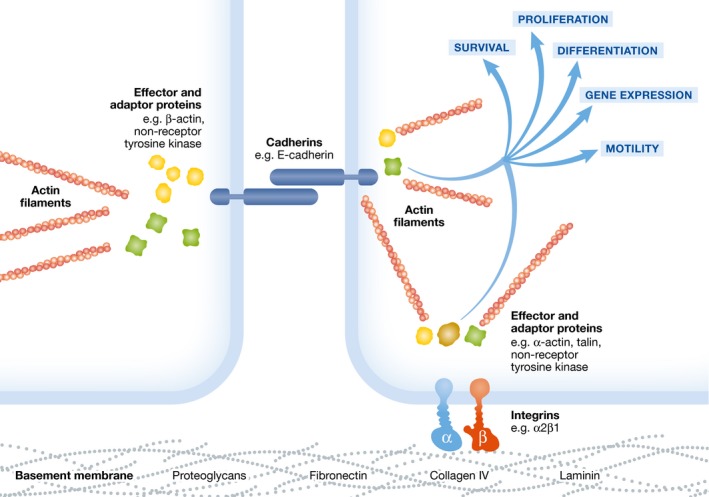
Cell–cell and cell–matrix interactions Physical interactions between the intestinal epithelium, adjacent cells, and the ECM provide pivotal signals for cell survival, proliferation, gene expression, differentiation, and motility. Adhesion molecules, such as integrins (e.g., α2β1) and cadherins (e.g., E‐cadherin) that attach to adjacent cells as well as to ECM proteins, mediate this. Adaptor and effector proteins provide linkage to intracellular actin filaments and can activate several signaling pathways, including non‐receptor tyrosine kinases.

Cell–matrix interactions are implicated in numerous cell functions, including differentiation, anoikis, proliferation, and gene regulation (Berrier & Yamada, 2007). This is accomplished through a set of membrane receptors, several of which are integrins (e.g., α2β1), that anchor the cells to the intestinal basement membrane (Lussier *et al*, 2000). Attachment to the intracellular cytoskeleton and activation of signaling pathways are achieved through recruitment of effector and adaptor proteins. This results in modification of anti‐apoptotic pathways, gene expression, cell differentiation, proliferation, and motility, as shown in Fig 2 (Lussier *et al*, 2000; Hofmann *et al*, 2007). In the absence of cell–matrix anchorage or cell–cell contact, epithelial cells undergo anoikis within hours (Hofmann *et al*, 2007).

**Figure 3:**
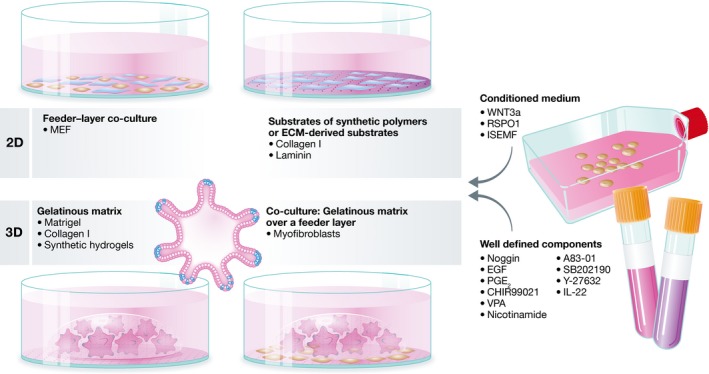
Culture protocols for ISCs Culture protocols for ISCs generally consist of two basic components: a support matrix and a growth medium. The support component can be in either 2D or 3D. 2D matrices are usually derived from feeder cells such as mouse embryonic fibroblasts (MEF), synthetic substrates or from ECM‐derived proteins, for example, collagen and laminin. 3D support matrices are usually in the form of gelatinous matrices, for example, Matrigel, collagen I, or synthetic hydrogels. Another approach is to use 3D co‐cultures, consisting of a gelatinous matrix over a feeder‐layer, for example, myofibroblasts. The growth medium often includes a conditioned medium, such as WNT3a, RSPO1, or intestinal subepithelial myofibroblasts (ISEMF), as well as fully defined growth factors, small molecules, and cytokines, for example, Noggin, EGF, nicotinamide, A83‐01, SB202190, PGE_2_, CHIR99021, VPA, and IL‐22.

Substantial efforts have been made to identify and optimize suitable matrices for stem cell cultures, particularly for culturing human pluripotent stem cells (hPSCs), which include induced pluripotent stem cells (iPSCs) and human embryonic stem cells (hESCs). Different culture protocols and support matrices are detailed in Fig 3.

Initial extracellular matrices (ECM) for culturing hPSCs were produced by feeder layers of lethally irradiated fibroblasts in enriched culture medium. Similarly, human colonic stem cells were recently successfully cultured on feeder layers of irradiated mouse embryonic fibroblasts over a Matrigel coating (Wang *et al*, 2015). Variability when using feeder layers, along with the prospect of denaturing or degrading peptides and proteins with sterilization techniques, as well as the potential risk for pathogen and xenogeneic transmission has led to the establishment of feeder‐free culture systems (Villa‐Diaz *et al*, 2013).

Corning^®^ Matrigel^®^ Matrix and BD Matrigel™ Basement Membrane Matrix are the most extensively used three‐dimensional (3D) support matrices for culturing ISCs. The extensive usage of Matrigel is attributed to its capacity to support long‐term growth of stem cells, while retaining the undifferentiated cell state (Hughes *et al*, 2010). It is a xenogeneic and proteinaceous matrix derived from mouse sarcoma cells, mainly composed of laminin, collagen IV, and entactin (Hughes *et al*, 2010). Its disadvantages include batch‐to‐batch variability, undefined composition, including varying amounts of sarcoma‐derived proteins, cytokines, and growth factors, along with the potential risk for pathogen transmission (Hughes *et al*, 2010). Such factors make Matrigel an ill‐suited culture platform for clinical application. Therefore, considerable efforts have been made to identify well‐defined matrices for both *in vitro* ISC expansion and their *in vivo* transplantation.

Collagen is an easily attainable connective tissue constituent, and common sources include fibroblasts cultured *in vitro*, as well as tissue extracts, such as human placenta. Different collagen formulations can be applied to sustain intestinal epithelial cell growth *in vitro* (Ootani *et al*, 2009; Yui *et al*, 2012). However, reduced budding has been reported when intestinal organoids are cultured in support matrices rich in collagen (Pastula *et al*, 2016), potentially due to increased mechanical rigidity. Recently, human ISCs isolated from small intestine were cultured to confluence on two‐dimensional (2D) monolayers on thin layers of bovine type I collagen and recombinant human laminin isotypes (Scott *et al*, 2016), although maintenance of the undifferentiated stem cell state was unclear at the protein level.

Another possible approach could be to utilize allogenic or xenogeneic tissues as ECM (e.g., small intestinal submucosa or urinary bladder matrix), which already are being used to culture other cell types in research and clinical settings. Tissues derived from natural sources are, however, restricted in their amplitude for modification, with inconsistencies related to the health and age of the donors (Fitzpatrick & McDevitt, 2015).

Biologic matrices suffer the disadvantages of batch‐to‐batch variability, relatively high manufacturing costs, limited scalability, and risk of pathogen contamination, motivating research on synthetic supportive matrices to overcome such issues. Synthetic matrices are chemically defined and malleable in terms of physiochemical and mechanical properties (Tong *et al*, 2015). Multiple types of 2D synthetic substrates have been used to culture hESCs. Further, certain isoforms of laminin and vitronectin, fibronectin, as well as other xeno‐free synthetic cell support matrices have successfully been used to support hPSC (Villa‐Diaz *et al*, 2013). Nevertheless, the conformation of vitronectin and laminin is sensitive to changes in temperature and pH, which limits their potential for long‐term usage (Tong *et al*, 2015).

3D matrices, as opposed to 2D ones, provide more space for the cells to grow, thereby reducing disadvantageous cell clustering (Lei *et al*, 2014). Furthermore, they efficiently provide physical and chemical gradients of importance for numerous cell functions, including differentiation and proliferation (Sant *et al*, 2010; Tong *et al*, 2015). Although simple collagen I 3D hydrogel matrices support ISCs, they have the disadvantage of low stiffness, limited long‐term stability, and batch‐to‐batch variability (Caliari & Burdick, 2016). 3D gels with compositions closer to the supportive matrix found *in vivo* or even mimicking axial gradients in connective tissue composition might be expected to provide improved viability and function of the cultured ISCs. Interestingly, fundamental ECM factors, such as mechanical properties and biochemical signals that regulate ISC colony and organoid formation, have recently been identified (Gjorevski *et al*, 2016). The efforts resulted in the formation of a mechanically dynamic polyethylene glycol (PEG) hydrogel, functionalized with RGD (Arg‐Gly‐Asp) peptides and controlled degradation kinetics, capable of expanding human small intestine and colorectal cancer organoids (Gjorevski *et al*, 2016). Hence, minimal PEG‐based hydrogels constitute a well‐defined alternative that might be applied to overcome the limitations of Matrigel in terms of clinical application of ISCs.

## Regenerative applications in IBD

Introduction of biologics like monoclonal antibodies against tumor necrosis alpha (TNF inhibitors) later followed by α4β7 anti‐integrins has revolutionized the management of IBD. However, despite these therapeutic advances about one‐third of patients with CD and one‐sixth of patients with UC still require surgical bowel resection within 5 years after diagnosis (Frolkis *et al*, 2013).

**Figure 4:**
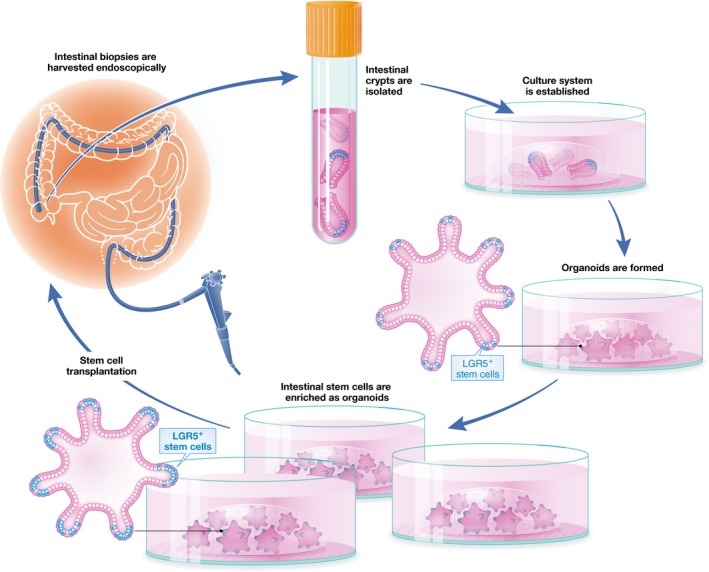
The course of human ISC harvesting to transplantation Human intestinal epithelium can be harvested endoscopically, and ISCs can subsequently be isolated and enriched *in vitro* as organoids. Organoids enriched in stem cells can then be transplanted back to the patient (or as a suspension of purified stem cells), thereby hopefully promoting mucosal healing.

Much like the majority of other medical therapies for IBD, TNF inhibitors and anti‐integrins act through immunomodulation. However, ISC transplantation constitutes a plausible alternative approach to accelerate mucosal healing. In fact, EGF is an effective treatment option for certain subtypes of IBD, possibly through its regenerative capabilities (Sinha *et al*, 2003). A schematic of the envisioned process, from harvesting of the ISCs to transplantation, is depicted in Fig 4.

Autologous transplantation may be performed in order to avoid the process of finding suitable cell donors, as well as circumventing the risk for tissue rejection and the need for further immunomodulatory therapy due to the procedure itself. ISCs would be harvested endoscopically from IBD patients with frequent and severe relapses during periods with remission, and then expanded and kept frozen until needed. An alternative approach could be to harvest ISCs from non‐inflamed areas during flares, or alternatively even from actively inflamed areas. However, excessive epithelial cell death can be observed in areas of active disease (Blander, 2016). Also, colonic organoids derived from patients with flaring UC have *in vitro* been shown to maintain an altered expression of genes associated with antimicrobial defense, absorptive and secretory functions, compared to healthy controls (Dotti *et al*, 2016). Additionally, lasting transcriptional changes in the affected epithelium have been observed in patients with UC despite remission (Planell *et al*, 2013). Although the consequences of such changes are unknown, for the purpose of transplantation it might be better to harvest ISCs from non‐involved epithelium. Another argument for this approach is that patients with IBD have ~1.5‐ to twofold increased risk of developing colorectal cancer (Beaugerie & Itzkowitz, 2015), presumably due to prolonged and remitting inflammation. When intestinal epithelial cells are harvested endoscopically, relatively few clones are afterward enriched *in vitro*. If these cells were to contain genetic mutations that predispose to malignancy, then transplantation might lead to risk of malignant transformation in a greater area of the intestine after engraftment. This important issue could, however, be addressed by screening for mutations known to be associated with colorectal cancer.

More than 160 susceptibility genes predisposing to IBD so far have been identified, including inflammatory bowel disease 5 (*IBD5*) and cadherin 1 (*CDH1*) that are associated with epithelial barrier function (Miner‐Williams & Moughan, 2016). Genetic susceptibility does not automatically lead to development of IBD, but transplantation of cells with a genetic susceptibility may potentially have implications on epithelial function even after a successful transplantation. Clearly however, more research on this matter is warranted.

ISCs should be transplanted as complete organoids or as small cell clusters with intact endogenous Notch stimulation to maintain stemness and delay anoikis. Successful engraftment would most likely require integrin activation to accomplish adherence to the ECM of the damaged mucosa, which in turn depends on extracellular divalent cations (Berrier & Yamada, 2007). In terms of delivery, endoscopic transplantation would intuitively be the simplest method, but enema could be an alternative route of administration—although the large volume needed in the latter case would greatly increase the need for cell expansion *in vitro*. Regardless of which method is chosen, a suitable delivery vehicle will be required to protect and sustain the cells in transit. Ideally, this should be fully defined and biocompatible, while allowing for *in situ* crosslinking and mucosal adhesion.

ISC transplantation may be able to spur the epithelial healing process, but for a majority of patients, it is unlikely that this would be successful as a monotherapy, as cells presumably will have difficulties engrafting during ongoing inflammation. Hence, concomitant immunomodulatory therapy will likely be needed to give the transplanted cells optimal conditions to re‐establish barrier integrity.

A crucial aspect of all cell‐based treatment strategies is to avoid inducing chromosomal changes that could lead to malignant transformation or other cell abnormalities. Epithelial stem cells grown *in vitro* can acquire a specific single nucleotide variant (SNV) signature differing from the somatic SNV signature seen *in vivo* in mice (Behjati *et al*, 2014). Long‐term cultivation of human ISCs has revealed a low level of genomic instability with a limited copy number and SNV instability for the first 100 days of continuous proliferation (Wang *et al*, 2015). Yet, a trend toward increasing SNV was observed as a function of passage number, but not involving reported driver genes in human cancer. However, a forthright chromosomal trisomy was noted after 200 days. It is possible that genetic changes acquired *in vitro* could increase the risk of introducing new mutations to the recipient of transplanted cells and could potentially increase the risk of neoplasia. Propagation of a sufficient amount of ISCs for regenerative application would, however, presumably require reasonably short culture duration. Accordingly, the risk of alterations in SNV signature and copy number could be minimized.

## Future perspectives

Alternative growth media compositions and culture protocols to increase the ISC yield are continuously being explored to allow for successful regenerative applications. This includes growth factors and small molecules that target the WNT pathway, such as PGE_2_ and CHIR99021, along with newly identified pathway targets such as IL‐22 and STAT3. Advancing regenerative applications of ISCs requires additional investigation to identify components affecting WNT, Notch, EGF, and BMP signaling that are apt for use in humans, preferably constituents that are already approved by FDA and EMA, or which demonstrate minimal or no toxicity.

Cell–cell and cell–matrix interactions have profound effects on cell phenotype and survival. The continuous development of alternative synthetic support matrices for ISCs is promising in terms of creating a suitable and indispensable substitute for Matrigel.

The number of ISCs attained *in vitro* is commonly estimated by determining *LGR5* expression levels. Yet, gene expression does not necessarily correlate to equivalent increases on protein level, and an increase in gene expression may reflect gene upregulation rather than increase in stem cell numbers. It would therefore be imperative to standardize how stem cell amplification is quantified *in vitro*. Modest quantities of LGR5 on the cell surface, along with the lack of selective antibodies with high affinity for human LGR5, hinder the effective quantification of ISC expansion. Still, the use of antibodies relies on applying specific surface proteins as surrogate markers for the intended cell population, although another approach is to use organoid forming capacity following single‐cell seeding as a functional assessment of stem cell numbers. Alternatively, single‐cell mRNA sequencing may be applied.

High levels of protein tyrosine pseudokinase 7 (PTK7) were recently reported to be a reliable surface marker for human colonic ISCs (Jung *et al*, 2015). While PTK7 does not exclusively stain ISCs, the cells with the highest PTK7 surface abundance are also the cells that demonstrate the highest capacity for organoid formation following single‐cell seeding. Hence, fluorescence‐activated cell sorting using antibodies for PTK7 can be applied to attain a purified pool of functional stem cells.

The establishment of culture techniques capable of guaranteeing quality and consistency are required for clinical usage of ISCs. In addition, improving cost‐effectiveness of the culture protocols would be favorable, as the sheer cost of the culture medium consisting of recombinant proteins can be a limiting factor.

Apart from IBD, ISC transplantation might in addition have implications in a wide range of other disorders of the gastrointestinal tract characterized by an impaired mucosal barrier function, including necrotizing enterocolitis, fistulas, NSAID‐induced damage, or gastroduodenal bleeding. In conclusion, the development of optimized protocols for culturing human ISCs can have a decisive impact on patient care in the future.

## Conflict of interest

J.M.K. and X.Y. hold equity in Frequency Therapeutics, a company that has an option to license intellectual property (IP) generated by J.M.K. and X.Y., and that may benefit financially if the IP is licensed and further validated. The interests of J.M.K and X.Y. were reviewed and are subject to a management plan overseen by their institutions in accordance with their conflict of interest policies. The remaining authors have no conflict of interest to declare.

**Pending issues**

- How do we maximize the yield of human ISCs *in vitro* in a way that is suitable for clinical application and how many cells are needed?
- What is the most appropriate method to quantify the yield of functional ISCs?
- What delivery vehicle and method should be used when performing transplantation?
- How do we assess engraftment efficiency after transplantation?
- Will cells continue to demonstrate a normal genotype and phenotype in the long term after transplantation?
